# Evaluation of the Feasibility and Acceptability of Perfect Fit, a Virtual Coach–Based mHealth Intervention for Smoking Cessation and Physical Activity in Adults: Mixed Methods Study

**DOI:** 10.2196/83456

**Published:** 2026-07-14

**Authors:** Milon H M van Vliet, Eline Meijer, Nele Albers, Kristell M Penfornis, Walter Baccinelli, Bouke L Scheltinga, Roxy A van Eersel, Niels H Chavannes, Anke Versluis, The Perfect Fit Consortium, Willem-Paul Brinkman

**Affiliations:** 1 Department of Public Health and Primary Care, Leiden University Medical Center, PO Box 9600, Leiden, 2300 RC, The Netherlands, 31 71 526 84 44; 2 National eHealth Living Lab, Leiden University Medical Center, Leiden, The Netherlands; 3 Department of Intelligent Systems, Delft University of Technology, Delft, The Netherlands; 4 Department of Communication and Cognition, Tilburg University, Tilburg, The Netherlands; 5 Department of Psychology, Unit Health, Medical, and Neuropsychology, Leiden University, Leiden, The Netherlands; 6 Netherlands eScience Center, Amsterdam, The Netherlands; 7 Biomedical Signals and Systems, University of Twente, Enschede, The Netherlands; 8 See Acknowledgments

**Keywords:** Physical activity, smoking cessation, virtual coach, conversational agent, chatbot, mHealth intervention, feasibility, acceptability, mixed methods

## Abstract

**Background:**

Mobile health (mHealth) interventions with virtual coaches offer scalable and potentially cost-effective solutions for health behavior change. However, these interventions commonly present challenges, such as limited personalization and insufficient grounding in evidence-based strategies. Perfect Fit (PF; Perfect Fit consortium), a personalized mHealth intervention with a text-based virtual coach, supports adults in quitting smoking and becoming more physically active. By combining innovative techniques, including sensor technology, end user involvement, and evidence-based strategies, PF aims to address common challenges faced by mHealth interventions, including those with virtual coaches.

**Objective:**

The study primarily investigated the feasibility and acceptability of PF. The secondary aim was to explore associations between sociodemographic, smoking-, and physical activity–related characteristics and the feasibility and acceptability outcomes. The third aim was to evaluate the feasibility of conducting the research study.

**Methods:**

A single-arm, pre-post, mixed methods study was conducted in the Netherlands with 100 adults who smoked. The intervention lasted approximately 16 weeks. Data were collected at baseline, during the intervention, and postintervention (4 months). Quantitative data included usage data and self-report questionnaires on feasibility, acceptability, and baseline characteristics. Qualitative data were gathered through postintervention semistructured interviews. Analyses included descriptive and inferential analyses, as well as the framework approach for the qualitative data.

**Results:**

PF usage varied considerably across participants (n=87). The mean satisfaction rating was 2.79 (SD 0.73; scale range 1‐4), and perceived usability had a median score of 67.50 (range 12.50‐87.50; scoring range 0‐100), indicating OK-to-good usability. The mean virtual coach acceptance rating was –0.27 (SD 1.30; scale range −3 to 3; n=77). Higher PF usage was associated with greater satisfaction, usability, and coach acceptance (all *P*≤.004). Frequent connection issues with the smartwatch were a disruptive factor. Qualitative findings (n=12) provided in-depth insights into PF’s feasibility and acceptability, encompassing both positive and negative experiences. For instance, some participants valued the virtual coach for its anonymity, low-threshold access, and the sense of control it offered, while others preferred a human coach for greater accountability. Suggested improvements included more varied content and enhanced adaptability of the coach to users’ input and personal situations. Exploratory analyses suggested that high PF users were older than moderate (*P*=.01) and low PF users (*P*=.05). Importantly, PF was perceived as similarly feasible and acceptable across socioeconomic groups (*P*>.05), aligning with one of the project’s goals. Finally, research procedures and recruitment strategies proved feasible.

**Conclusions:**

PF shows potential as an accessible and inclusive strategy for multiple health behavior changes, contributing to public health. Findings highlight areas for improvement and can guide the future development of virtual coach interventions.

## Introduction

The development of mobile health (mHealth) interventions has increased noticeably over the past 30 years [1,2], offering promising solutions in terms of reach, accessibility [3], and scalability [2,4]. A systematic review of reviews has shown that mHealth interventions can support individuals in adopting healthier behaviors [5]. This is especially relevant for high-risk behaviors such as smoking and insufficient physical activity (PA), which are major contributors to chronic diseases and premature mortality [6-8] and the primary targets of the intervention evaluated in this study. In 2021, approximately 17% of the global adult population smoked [8], and in 2022, 31% did not meet PA recommendations. Moreover, unhealthy behaviors often co-occur [9,10], especially among individuals with lower literacy or in vulnerable positions. These individuals also tend to experience poorer health outcomes and reduced life expectancy [6,11-14]. Given the high prevalence and health burden of smoking and insufficient PA, these behaviors are key public health concerns [6].

Encouraging smoking cessation and PA promotion simultaneously may be particularly beneficial, as they can reinforce each other [15-17]. PA can help reduce nicotine cravings [15] and alleviate withdrawal symptoms [16], while quitting smoking improves fitness, potentially encouraging more PA [17]. However, sustained behavior change is difficult to achieve without effective support. Studies show that the likelihood of successfully quitting smoking is around 3 times higher with professional support compared to no support [13,18]. Similarly, PA interventions incorporating behavioral support elements (eg, feedback on performance) can effectively promote both the initiation and maintenance of PA [19]. This highlights the need for effective support to facilitate lasting behavior change.

Due to the ongoing health care crisis, professional support is increasingly limited. Health care demand exceeds supply [20], and mHealth interventions could help alleviate this burden by providing scalable [2,21], remote [4], and potentially cost-effective [5] smoking cessation and PA promotion support [5]. They can empower individuals to take greater control of their health, improve health outcomes, and potentially reduce health care use [22]. Additionally, mHealth interventions may lower barriers related to fear of stigmatizing interactions when seeking support, often experienced by people who smoke [21]. Finally, they enable just-in-time adaptive interventions, which adjust delivery based on the time of day and integrate into users’ daily lives [4,23]. Hence, mHealth interventions are seen as a promising way to meet growing health care demands.

Although promising, mHealth interventions also have disadvantages. Compared to human support, they are often experienced as less personal and tailored [24] and can evoke a reduced sense of personal accountability [21], which can contribute to low user engagement and adherence [4]. Integrating virtual coaches into mHealth interventions may help overcome these limitations by simulating human support while maintaining digital delivery advantages. Virtual coaches are AI conversational agents that mimic human interactions through text, speech, or both [1,25,26]. They offer interactive, personalized support, which may enhance adherence and engagement compared to more static mHealth interventions [27]. Reviews of virtual coach interventions for smoking cessation [21,25] and PA promotion [1] reported positive outcomes regarding effectiveness, acceptability, and user experience. However, these findings should be interpreted with caution due to study heterogeneity and underpowered studies. While these findings highlight the potential of virtual coaches, further research is needed to address key challenges and knowledge gaps.

The literature on mHealth interventions with virtual coaches identifies key challenges, including a lack of theory-based development; limited and mixed evidence on long-term engagement (eg, due to nonstandardized measurement methods) [1,4,25,28]; and digital inclusion problems [2,20,21,24]. Several strategies have been proposed to address these challenges. First, it is crucial to base interventions on behavior change techniques [29], theories, or models [1,4,14]. Second, personalizing content and timing may increase relevance [27], a sense of autonomy [25], and engagement [4,27,28]. In PA interventions, wearables can further support this personalization by enabling real-time feedback [30]. Third, prioritizing simple and user-friendly content can increase engagement [4,14,31]. Fourth, incorporating relational strategies, such as motivational interviewing [28] or emoji [3] to mimic human-like interactions, can support long-term behavior change [25,32]. Finally, interdisciplinary collaboration [4,21] and early end user involvement in development [14,20,22,31,33] can improve research and intervention quality. Incorporating these strategies is vital, as low engagement hinders the feasibility, acceptability, and, ultimately, effectiveness of interventions [25,27,28].

In addition to these challenges, the literature highlights several knowledge gaps, including limited research on the feasibility and acceptability of virtual coaches [1,5]. While effectiveness is crucial, long-term adoption also depends on factors such as ease of use, trust in the technology, and user satisfaction, making it essential to understand user needs and experiences [24-26,33,34]. To address this knowledge gap, qualitative and mixed methods designs can help to explore facilitators, barriers, and limitations [14,26,33]. Another knowledge gap is the limited understanding of who benefits most from virtual coaches and under which circumstances [1,25,26]. Such insights could support tailored intervention development [4], improve feasibility and acceptability across diverse populations [26], and help identify target groups [5]. Particular attention is needed for individuals with lower digital skills, eHealth literacy [20,21,24], and socioeconomic positions (SEP) [1,2,14]. Although mHealth interventions have the potential for broad scalability [2], many remain inaccessible to these populations [20,22,31]. To avoid exacerbating health disparities [20-22], virtual coaches should be designed with accessibility in mind [2,14]. This includes early end user involvement [14,20,22,31,33,35] and tailoring interventions to users’ digital skills and eHealth literacy [20,22]. It is also key to examine how individual characteristics, such as age, SEP, and eHealth literacy, influence feasibility and acceptability to identify who benefits most [33].

With an interdisciplinary consortium, we developed Perfect Fit (PF; Perfect Fit consortium) [36], a smartphone-based mHealth intervention with a virtual coach that provides real-time, personalized, text-based, and visual feedback to support smoking cessation and PA promotion. PF was specifically designed to leverage the assumed synergy between these behaviors. It also addresses common challenges and knowledge gaps in mHealth interventions, including those with virtual coaches. For instance, it includes behavior change techniques (eg, goal-setting) [29] commonly used in smoking cessation [37] and PA promotion [38], as well as behavioral theories, namely identity theories [39-41] and the Relapse Prevention Model [42]. Additionally, PF incorporates sensor-based personalized feedback using a smartwatch and was developed iteratively with end users [43].

This study presents the results of the real-world evaluation of PF, using a single-arm, pre-post, mixed methods design. The first aim is to investigate the feasibility and acceptability of PF. The second aim is to explore associations between baseline characteristics (ie, sociodemographic-, smoking-, and PA-related characteristics) and the feasibility and acceptability outcomes. This will provide insight into factors influencing intervention success and PF’s suitability for different individuals. The third aim is to investigate the feasibility of conducting the study itself. Many mHealth studies face high dropout rates [21] and difficulties in recruiting participants, particularly those with lower SEP [14]. Examining these factors can inform effective recruitment and retention strategies for future research. By using a mixed methods approach, we gain insight into PF’s overall potential, participant experiences, and contextual factors. This will help identify strengths and areas for improvement, ultimately informing future development and evaluation.

## Methods

### Study Design

This paper reports on a larger study conducted in the Netherlands, in which PF was evaluated using a single-arm, pre-post, convergent mixed methods design [43]. This paper focuses on the feasibility and acceptability of PF, as well as the feasibility of conducting the study itself. The data relevant to this paper were collected between August 2023 and June 2024. Findings on short- and long-term preliminary effectiveness will be reported elsewhere. This study is reported in line with the Good Reporting of A Mixed Methods Study (GRAMMS) checklist [44] (Checklist 1).

To assess the feasibility and acceptability of PF and the study itself, data were collected at baseline, during the intervention (ie, user log data and sensor data), and at postintervention (4 months after baseline). The quantitative component included (1) self-report questionnaires on baseline characteristics, feasibility and acceptability of PF, and virtual coach acceptance; (2) app-generated usage data; and (3) data on study procedures documented in a researcher’s log. Self-report questionnaires comprised both self-developed and adapted items, as well as validated instruments, including the eHealth Literacy Questionnaire (eHLQ) [45,46], the Fagerström Test for Nicotine Dependence (FTND) [47], the Godin-Shephard Leisure-Time PA questionnaire (GSLTPAQ) [48,49], and the System Usability Scale (SUS) [50]. The qualitative component consisted of semistructured interviews exploring the feasibility and acceptability of (1) PF, (2) the virtual coach, and (3) the study procedures. The study was approved by the scientific committee of the Department of Public Health and Primary Care at Leiden University Medical Center (approval number: WSC-2023‐24). Full study details are available in the published protocol [43]. Deviations from the protocol are reported below.

### Patient and Public Involvement

Patient and public involvement (PPI) activities were conducted during the development of PF and the execution of this study. Multimedia Appendix 1 provides a description of the PPI aims, methods, results, and reflections, reported according to the revised version of the Guidance for Reporting Involvement of Patients and the Public (GRIPP2) short-form checklist [51] to ensure transparency and comprehensiveness. Additional details on PPI activities can be found in the published article on the development of PF [36].

### Participants and Recruitment

Participants were recruited using a combination of online and offline strategies. Examples include social media posts and advertisements, newsletters or websites of affiliated organizations (eg, health care insurers), a news item in a local newspaper, and outreach via the networks of the PF research team and advisory panel. Recruitment started mid-August 2023, and the final participant was included on February 5, 2024. Sample size estimation is discussed in the study protocol [43].

Participants were eligible if they (1) were aged ≥18 years, (2) smoked daily, (3) intended to quit smoking within 6 weeks, (4) could walk pain-free, (5) could understand and read Dutch (at least B1 level), and (6) owned a smartphone with Internet access. To examine PF in high-risk individuals, at least 50% of participants were required to be at higher cardiovascular risk according to the Dutch general practice guideline [52]; that is, women aged ≥55 years or men aged ≥50 years. Additionally, at least 75% of participants had to reside in the Leiden region. Exclusion criteria included (1) enrollment in smoking cessation treatment at intervention start at the time of eligibility screening, (2) major lower extremity surgery in the past year, (3) use of antipsychotics or diagnosis of a serious psychiatric illness (eg, schizophrenia/psychosis or major depression), and (4) pregnancy.

For the qualitative interviews, we aimed to recruit around 10‐15 participants to achieve data saturation [53]. A heterogeneous subsample was sought, with variation primarily in PF usage level, as well as in gender, age, SEP, and success in changing health behavior.

### Intervention

PF is an evidence-based, personalized mHealth intervention with a virtual coach that supports individuals in quitting smoking and increasing PA. The intervention was expected to last around 16 weeks but could be adjusted to individual needs. The virtual coach, “Sam,” guided users through 3 phases, including a preparation phase, an execution phase, and a closing dialog. Communication consisted of chat messages enriched with emoji, images, and animated informational videos, and users received links to relevant external sources. The coaching system allowed for both system- and user-initiated conversations, with a mix of constrained (preset responses) and unconstrained (free-text) input. A smartwatch (Garmin Forerunner 55; Garmin Ltd) [54] measured PA, enabling the coach to provide personalized PA goals and feedback. Users interacted with the coach via a smartphone app with chat functionality (ie, the NiceDay app; NiceDay Healthcare Nederland BV; [55], originally developed for remote therapy), and users installed 2 additional apps to enable a connection between the smartwatch and the coaching system.

In the preparation phase, users were encouraged to complete activities and dialogs that prepared them for quitting smoking and increasing PA. These dialogs covered topics such as medication and nicotine replacement therapy and self-monitoring of current behavior. Users were also guided in selecting a quit date and formulating a specific, measurable, achievable, relevant, and time-bound (SMART) long-term PA goal through the coach-initiated goal-setting dialog. After this dialog, the coach provided daily short-term PA goals (ie, step goals) based on previous PA measured via the smartwatch, along with feedback on whether the goal was met. The self-selected quit date (within a predefined range) then marked the start of the execution phase, during which the coach provided support through activities facilitating behavior change and weekly reflections on progress. The closing dialog was the final conversation in which the coach encouraged users to review their progress and achievements and develop a relapse prevention plan. PF included 21 coach-initiated core components considered important for supporting behavior change and forming the overall intervention structure. These consisted of 7 preparation phase dialogs or videos, the introduction video for the execution phase, the weekly reflection dialog occurring 12 times, and the closing dialog. In addition, users could initiate optional components at any time, including dialogs (eg, a high-risk situation and relapse dialog for difficult moments) and 25 short activities (eg, positive self-talk healthy eating tips), which could be repeated as often as desired to enhance engagement and personalization. Further details on the development of PF [36], the intervention components and features [43], the technical architecture [56], and the open-source code of the system [57] are described elsewhere.

### Procedures

After consent, an onboarding procedure was initiated. Participants received the smartwatch and information materials. Specifically, participants received a video explaining the study and intervention, an installation booklet with instructions on app installation and smartwatch-coach connection, and an information booklet summarizing key instructions and tips for interacting with the coach. The latter also served as a workbook, offering space for notes. After receiving the materials, participants had a meeting with a researcher (via video call or telephone) to verify app installation, coach connection, and smartwatch use. During the intervention, participants could contact the research team via email or phone for technical support. A detailed participant timeline is described in the study protocol [43].

#### Quantitative Data Collection

Participants received a link to the online baseline questionnaire (T0) during the week of the onboarding meeting and the postintervention questionnaire (T1) 16 weeks later, at the expected end of the intervention. To maximize completion, researchers reminded participants via email and phone calls. Participants could keep the smartwatch if they completed at least 80% of the questionnaires of the larger study.

#### Qualitative Data Collection

A subsample of participants who had given prior consent to be contacted for an interview was invited at T1. Interviews were conducted online or in person (at Leiden University Medical Center), lasted approximately one hour, and were carried out by 2 medicine master’s students trained in interviewing. In order to prevent social desirability bias, the interview schedule consistently focused on both positive and negative aspects of PF in general and its specific components.

### Outcomes

Only measures relevant to the present paper are reported below. The interviews conducted at T1 were used to assess both primary and secondary qualitative outcomes (eg, experiences with PF and research participation). Full details are described in the protocol [43].

#### Baseline Variables

The following variables were assessed at baseline (T0) and were used to describe the sample and explore associations with the primary outcomes.

##### Participant Characteristics

Background characteristics were assessed using self-developed items and included gender, age, educational level (as a measure for SEP) [58], and the presence of any physical or mental (chronic) conditions.

##### eHealth Literacy

Assessed using 5 of the 7 scales of the Dutch eHLQ: (1) using technology to process health information (Cronbach α=0.77; McDonald *ω*=0.81); (2) understanding of health concepts and language (Cronbach α=0.77; McDonald *ω*=0.84); (3) ability to actively engage with digital services (Cronbach α=0.83; McDonald *ω*=0.88); (4) feel safe and in control (Cronbach α=0.83; McDonald *ω*=0.87); and (5) are motivated to engage with digital services (Cronbach α=0.81; McDonald *ω*=0.85) [45,46]. The remaining 2 scales were not included, as they were not considered relevant to our study. Each scale consists of 5 items rated on a 4-point scale from 1 (strongly disagree) to 4 (strongly agree).

##### Intention to Quit Smoking

Assessed using a single item, *“*Which of the following plans best applies to you? I intend to…” [59]. Answer categories were (1) quit within the next month, (2) quit between 1 and 6 months from now, (3) quit sometime in the future, beyond 6 months, or (4) not planning to quit.

##### Smoking Behavior and Physical Nicotine Dependence

Assessed using a Dutch translation of the 6-item FTND (eg, “Do you smoke more in the morning than during the rest of the day?*”*) [47,60]. Total scores were calculated according to standard FTND scoring, resulting in a total score ranging from 0 to 10, with higher scores indicating higher nicotine dependence.

##### Using E-Cigarettes

Participants were asked, using self-developed items, whether they used e-cigarettes. If they responded “yes,” they were subsequently asked whether they used e-cigarettes with or without nicotine.

##### Intention to Become Sufficiently Physically Active

Assessed using a single item, *“*Which of the following plans best applies to you? I intend to…” [59]. Answer categories were (1) become sufficiently physically active within the next month, (2) become sufficiently physically active between 1 and 6 months from now, (3) become sufficiently physically active sometime in the future, beyond 6 months, or (4) not planning to become sufficiently physically active. Before this question, the definition of PA (ie, exercise, but also physical activities like walking to the supermarket) and the World Health Organization (WHO)–recommended PA guidelines [7] were explained.

##### Self-Reported Level of PA

Assessed using a Dutch translation of the 3-item GSLTPAQ [48,49]. Participants were asked to report, on average, how many times during a typical week they engaged in more than 15 minutes of (1) strenuous exercise (eg, running and soccer), (2) moderate exercise (eg, brisk walking and badminton), or (3) mild exercise (eg, easy walking yoga). Example activities for each intensity level were provided. Total scores were calculated according to standard GSLTPAQ scoring. A total score of 24 or more was defined as “active,” a score between 14 and 23 as “moderately active,” and a score below 14 as “insufficiently active” [48].

### Feasibility and Acceptability of PF and the Virtual Coach

To investigate the feasibility and acceptability of PF, the following primary outcomes were assessed [43].

#### PF Usage (During Intervention and T1)

Objective intervention usage data collected through the app throughout the intervention duration included (1) the number of days used (ie, from the first to the last recorded activity in the app), and (2) the number of completed coach-initiated core intervention components (ie, 21 in total). Components were only logged as completed if finished in full, providing a conservative estimate of usage. As part of the T1 questionnaire, participants also self-reported how frequently they interacted with the virtual coach during PF using a self-developed item.

A deviation from the study protocol [43] was that we did not analyze the self-reported intervention completion item. Due to the personalized intervention duration and content, the definition of “completion” was somewhat subjective. Therefore, app usage data, which objectively tracked completed components, was considered more reliable and also correlated significantly with self-reported completion.

#### Satisfaction With PF (T1)

Assessed using 2 self-developed items that were rated on Likert scales from 1 to 4 (Spearman-Brown *r*=0.75, indicating sufficient overlap to allow averaging the scores on the 2 items): *“*How satisfied are you with the amount of support you received?” and *“*Would you use the Perfect Fit program again, if needed?” Higher average scores reflected greater satisfaction.

#### Usability of PF (T1)

Assessed using the 10-item SUS [50] (Cronbach α=0.84; McDonald *ω*=0.90). Responses were given on a 5-point Likert scale from 1 (strongly disagree) to 5 (strongly agree; eg, “I think the Perfect Fit program is unnecessarily complex”). Standard SUS-scoring was used, resulting in total scores ranging from 0 to 100, with higher scores indicating greater usability.

#### Acceptance of the Virtual Coach (T1)

Assessed with 6 items adapted from Provoost et al [61] covering satisfaction, usability, willingness to continue interaction, relationship, preference for a human coach versus coach Sam, and adherence to advice from coach Sam [62] (Cronbach α=0.81; McDonald *ω*=0.90). Items were scored on a 7-point Likert scale from −3 to 3 (eg, “How satisfied were you with Coach Sam?”). Item 2 was reverse-scored, so that higher average scores indicated more positive attitudes.

#### Qualitative Data From Semistructured Interviews (T1)

Qualitative feedback regarding participants’ experiences with PF, the virtual coach, and the smartwatch complemented the quantitative data (see protocol for the interview protocol [43]).

### Sensor Data Issues (During Intervention)

During the study, we observed frequent issues with the connection between the smartwatch and the virtual coach. These issues caused the coach to communicate inaccurate step counts and goals and triggered repeated notifications from the coach to the user about connection issues. The extent and nature of the sensor data issues were investigated, as they may have negatively impacted PF’s feasibility and acceptability. This was analyzed as an exploratory outcome and was an addition to the original protocol [43].

### Study Feasibility

The following secondary outcomes were used to assess the feasibility of conducting the study: (1) recruitment, response, and consent rates, recorded by the researchers in a participant screening and inclusion log throughout the study; (2) recruitment strategies, assessed at T0 via a self-report item asking participants how they were informed about the study (eg, via social media); (3) study adherence, monitored by the researchers from onboarding until T1 (ie, those who withdrew before completing onboarding were categorized as withdrawals rather than dropouts); and (4) qualitative data from semistructured interviews at T1, capturing participants’ experiences with research participation (see protocol for the interview protocol [43]).

### Data Analysis

#### Quantitative

Most data preparation and analyses were performed in SPSS (version 29.0; IBM Corp), and RStudio (version 2024.04.2+764; PBC) was used for calculating McDonald omega and for preparing and analyzing PF usage and step count data. Details on data preparation, including age and SEP coding, winsorizing [63] of extreme outlying PA values, dichotomization of variables for exploratory analyses, and sensor data processing are provided in Multimedia Appendix 2.

Descriptive statistics (eg, mean, SD, and frequencies) were used to summarize baseline characteristics of the study sample. Chi-square tests, independent samples *t* tests, and Mann-Whitney *U* tests were used for dropout analyses.

To address the primary and tertiary study aims—investigating the feasibility and acceptability of PF, acceptance of the virtual coach, and feasibility of conducting the study—descriptive analyses were conducted.

To address the secondary aim—exploring associations between baseline characteristics and the feasibility and acceptability outcomes—exploratory one-way ANOVAs, Kruskal-Wallis tests, Mann-Whitney *U* tests, chi-square tests, and Pearson and Spearman correlations were performed. Post hoc pairwise comparisons were conducted with Tukey honestly significant difference (HSD) corrections for ANOVAs and Bonferroni corrections for Kruskal-Wallis tests.

For the exploratory sensor data issue analyses, descriptive statistics were used. Associations between days with step count data and weekday, phone operating system, and PF usage group were explored with a Pearson chi-square test, a Mann-Whitney *U* test, and a Kruskal-Wallis test.

#### Qualitative

All semistructured interviews were audio-recorded, pseudonymized, and transcribed verbatim. Qualitative data were analyzed using the framework approach [64,65] in ATLAS.ti (version 23.2.3.27778; ATLAS.ti Scientific Software Development GmbH). The first transcript was independently coded by the 2 master’s students who had conducted the interviews and by MHMvV, resulting in an initial coding scheme. The students then independently coded and discussed the second and third transcripts, refining the coding scheme when discrepancies arose. The remaining transcripts were each coded by one student, with the coding scheme further adjusted as needed. All revisions were discussed with MHMvV, RAvE, and EM. Data interpretation and theme generation were carried out by MHMvV in collaboration with EM and AV and were guided by the research aims, with consideration of the corresponding quantitative outcomes. Interview quotations were labeled with participant identifiers according to PF usage category (L=low, M=moderate, and H=high) and an individual interview number. Quotations were translated from Dutch to English with care to preserve the original meaning.

#### Data Triangulation

The analysis followed a convergent mixed methods approach. Quantitative and qualitative data were first analyzed separately using applicable analytical methods. Qualitative codes were derived from the interview data and organized within categories aligned with the study aims and quantitative outcomes. Subsequently, findings from both data sources were compared and integrated for each outcome. Results are therefore presented per outcome, combining quantitative results with qualitative findings to illustrate convergence and divergence and to provide additional context to the interpretation of the data.

### Ethical Considerations

This study has been approved by the scientific committee of the Department of Public Health and Primary Care at Leiden University Medical Center (approval number: WSC-2023‐24). The Medical Research Ethics Committee Leiden, The Hague, and Delft reviewed the study proposal and provided a declaration of no objection, indicating the research does not fall under the Dutch Medical Research with Human Subjects Law (nWMO; approval number: N23.045 METC-LDD). Interested individuals received a digital information letter and a link to the online screening questionnaire (hosted in secure software, Castor electronic data capture) [66]. Eligible participants were included after signing an online informed consent form and were informed that they could withdraw from the study at any time for any reason. Verbal informed consent was obtained and audio-recorded before each interview. Participants received a €25 (based on the conversion rate of 1.0786 between August 2023 and June 2024, this amount is equivalent to approximately US $26.97) gift voucher for the interview.

## Results

### Sample Characteristics

**Figure 1.**
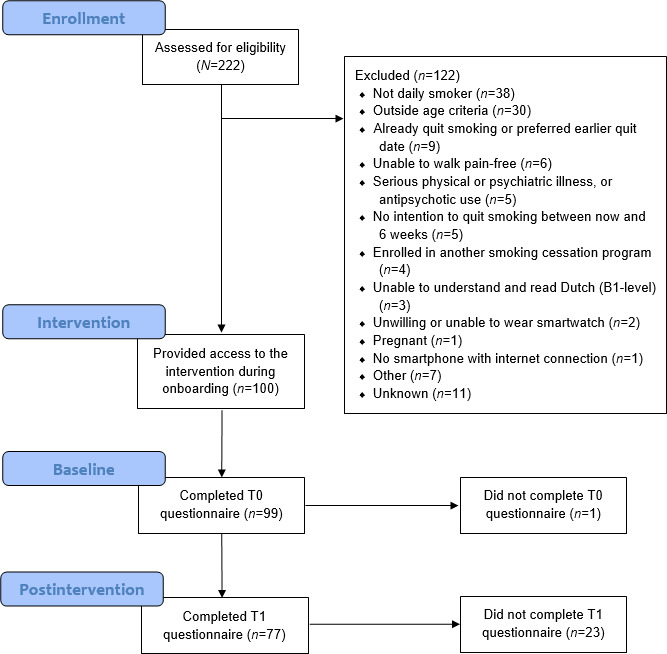
Adapted CONSORT (Consolidated Standards of Reporting Trials) participant flow diagram (adapted from Hopewell et al [67]).

The participant flow diagram is presented in Figure 1 [67]. One hundred participants were given access to PF during onboarding. One participant dropped out before completing the baseline questionnaire, resulting in 99 participants who started PF and completed the baseline questionnaire.

Baseline sociodemographic, smoking- and PA-related characteristics for the total sample (n=99) and T1 completers (n=77) are presented in Table 1. Of the 99 participants, 58.6% (n=58) were female, and the mean age was 51.96 (SD 14.08) years, ranging from 19 to 86 years. The distribution of SEP was comparable to the general Dutch population [68]. Median scores on the 5 eHealth literacy scales ranged from 2.80 to 3.00 on the 4-point scale. Regarding smoking-related characteristics, the majority of participants (n=77, 77.8%) intended to quit smoking within one month. At baseline, participants smoked an average of 15 cigarettes per day and reported low-to-moderate nicotine dependence [47,69]. Regarding PA-related characteristics, 54.5% (n=54) of the participants intended to be sufficiently physically active within one month. The median GSLTPAQ score suggests that at least half of the participants were already sufficiently active at baseline. However, the GSLTPAQ data were highly skewed and included extreme outliers; therefore, absolute values should be interpreted with caution. Dropout analyses showed that none of the baseline characteristics were significantly associated with study adherence at T1.

A total of 12 participants actively withdrew (via email or phone) from both the intervention and the study (ie, full dropouts), and 7 participants discontinued the intervention but continued completing study questionnaires. Reasons for dropout included: (mental) health problems (n=4), persistent technical issues with the smartwatch and/or app (n=4), dissatisfaction with the smartwatch functionality or intervention content (n=4), personal circumstances (n=1), a preference for human support over virtual coaching (n=1), smoking relapse and stress experienced due to the PA component of the intervention (n=1), having quit smoking and therefore no longer needing the intervention (n=1), and unknown reasons (n=3).

**Table 1.** Baseline characteristics of the total sample and of postintervention completers.

Baseline characteristics	Baseline (T0) completers (total sample), n=99	Postintervention (T1) completers, n=77
Gender, n (%)		
Female	58 (58.6)	42 (54.5)
Male	40 (40.4)	34 (44.2)
Other	1 (1.0)	1 (1.3)
Age (years), mean (SD)	51.96 (14.08)	51.29 (13.71)
SEP^a^, n (%)		
Low	20 (20.2)	18 (23.4)
Middle	44 (44.4)	31 (40.3)
High	34 (34.3)	27 (35.1)
Prefer not to say	1 (1.0)	1 (1.3)
Physical or mental (chronic) conditions, n (%)		
No condition	69 (69.7)	56 (72.7)
Physical condition/conditions	21 (21.2)	15 (19.5)
Mental condition/conditions	5 (5.1)	3 (3.9)
Both physical and mental condition/conditions	4 (4.0)	3 (3.9)
eHealth literacy (eHLQ)^b^, median (range)		
Scale 1: using technology to process health information	2.80 (1.80‐4.00)	3.00 (1.80‐4.00)
Scale 2: understanding of health concepts and language	3.00 (1.60‐4.00)	3.00 (2.00‐4.00)
Scale 3: ability to actively engage with digital services	3.00 (1.80‐4.00)	3.00 (1.80‐4.00)
Scale 4: feel safe and in control	3.00 (1.60‐4.00)	3.00 (1.60‐4.00)
Scale 5: motivated to engage with digital services	3.00 (1.60‐4.00)	3.00 (1.80‐4.00)
Intention to quit smoking, n (%)		
Between now and 1 month	77 (77.8)	60 (77.9)
Between 1 and 6 months	22 (22.2)	17 (22.1)
In the future, but not within 6 months	0 (0.0)	0 (0.0)
Number of cigarettes smoked a day, median (range)	15.00 (2.00‐60.00)	16.00 (2.00‐60.00)
Nicotine dependence (FTND)^c^, mean (SD)	4.40 (2.23)	4.38 (2.27)
Using e-cigarettes with nicotine, n (%)		
No	90 (90.9)	69 (89.6)
Yes	9 (9.1)	8 (10.4)
Intention to become sufficiently physically active, n (%)		
Between now and 1 month	54 (54.5)	41 (53.2)
Between 1 and 6 months	43 (43.4)	35 (45.5)
In the future, but not within 6 months	2 (2.0)	1 (1.3)
Never	0 (0.0)	0 (0.0)
Level of PA^d^ (GSLTPAQ)^e^, median (range)	24.00 (0.00‐79.00)	25.00 (0.00‐79.00)

a SEP: socioeconomic position.

b eHLQ: eHealth Literacy Questionnaire.

c FTND: Fagerström Test for Nicotine Dependence.

d PA: physical activity.

e GSLTPAQ: Godin-Shephard Leisure-Time Physical Activity Questionnaire.

### Qualitative Interview Sample

Twelve participants took part in the postintervention individual interviews, forming a heterogeneous sample, including 3 low PF users, 5 moderate users, and 4 high users. Seven of the 12 participants were male, the average age was 54.8 (range 35‐77) years, and SEP levels varied (see Table S1 in Multimedia Appendix 2 for detailed characteristics).

### Primary Outcomes

#### Feasibility and Acceptability PF

##### Usage of PF

Twelve of the 99 participants had missing PF usage data, likely due to dropping out before entering their participant code in the app or entering it incorrectly, which prevented linkage between their app usage data and questionnaire responses.

Participants with available PF usage data (n=87) completed on average 54.7% of the 21 core intervention components (mean 11.49, SD 5.89). Six participants completed all components. The median PF usage duration was 107 days (range 4‐202), which closely approximates the expected intervention period of 16 weeks (ie, 112 days). Participants completed a median of 4 optional short PF activities (range 0‐46, as all 25 available activities could be repeated). Twelve participants completed none. Participants’ self-reported frequency of weekly coach interaction is presented in Multimedia Appendix 2. A significant positive correlation was found between the number of completed core components and optional activities (Spearman ρ=0.638*; P*<.001). For subsequent exploratory analyses, usage was categorized into 3 groups (low, moderate, and high) based on the number of core components completed. See Table S2 in Multimedia Appendix 2 for details on this categorization and the corresponding distribution of optional activities completed within each group.

Exploratory analyses (n=77) revealed significant differences between PF usage groups in the other primary outcomes: satisfaction with PF (*F*_2, 74_=6.09; *P*=.004; *η^2^*=0.14), usability of PF (*H*_2_=11.16; *P*=.004), and acceptance of the virtual coach (*F*_2,74_=8.59; *P*<.001; *η^2^*=0.19). Post hoc tests showed that high PF users reported significantly higher scores than moderate users on all 3 outcomes. Additionally, high users reported significantly higher scores on the acceptance of the coach than low users.

Qualitative data provided insight into reasons for lower usage of PF. Some participants reported that, after a while, they found PF or the virtual coach too intense, repetitive, or even irritating, which led to lower usage:

*In the last few weeks, I used Coach Sam a bit less because I found it quite intense.*

[M2, participant who was still smoking and increased PA at T1]

Others expressed a growing desire for autonomy, leading them to reduce their interaction with the coach as they gained confidence in managing their behavior change independently:

*Over time, I noticed I got used to it […] and started looking at it less. In the beginning, I used it a lot, but gradually I started thinking. ‘I’ll do this myself now.’*

[H2, abstinent and decreased PA at T1].

##### Satisfaction With and Usability of PF

At postintervention, the mean satisfaction rating was 2.79 (SD 0.73; n=77) on a scale from 1 to 4. Perceived usability had a median score of 67.50 (range 12.50‐87.50), indicating OK-to-good usability [70].

Qualitative interview data revealed that participants expressed both positive and negative experiences regarding satisfaction with and usability of PF, which are summarized in Table 2. Some features were experienced differently by participants, such as perceptions of how enjoyable and motivating PF was and the perceived variation in short optional activities. Issues with the connection between the smartwatch and the coach were often mentioned and likely impacted PF’s feasibility and usability (see Results, Exploratory analyses section, “Sensor data issues”).

**Table 2.** Perceived strengths and points for improvement of Perfect Fit, with illustrative quotations from the qualitative interviews, grouped under descriptive labels reflecting recurring topics in participants’ responses*.*

Strength	Point for improvement
General experience	
Useful, comprehensive, and informative *“*I really think it’s a good product you’ve created, and I genuinely believe it’s useful and effective for people seeking that kind of support. I do hope that […] such a product could become available on the market for smoking cessation and increasing physical activity.” (H1^a^, abstinent and decreased PA^b^ at T1^c^)	—^d^
Enjoyable^e^ *“*When it worked properly [without technical issues], I enjoyed using Perfect Fit.” (H3, abstinent and increased PA at T1)	Not sufficiently enjoyable/engaging *“*It worked, so I thought it was a good program. But I didn’t find it very engaging. I wasn’t looking forward to it like, ‘Oh great, I get to use the program again soon’, or anything.” (M5, abstinent and increased PA at T1)
** **Motivating *“*It’s a good initiative. I think it stimulates people. […] Those videos were good, also the questions that were asked and the occasional tips you got. […] Like, ‘What is this really doing to you?’. It actually says what you already know. We all know it, but we push it aside, and those things were confronting again. Like, ‘Oh right, that could indeed be a consequence’ – but it was told in a pleasant and friendly way.” (H2, abstinent and decreased PA at T1)	Not sufficiently motivating *“*In general, I found it very interesting because you’re really working on your own development. You really start thinking about it. And you actually do move more and smoke less. Except at the end of the program, because maybe you become a bit more complacent – or maybe coach Sam just didn’t provide the right trigger. I thought that was a pity. But I do think it’s very personal.” (M2, smoking and increased PA at T1) *“*It didn’t motivate me enough to quit smoking.” (L1, smoking and increased PA at T1)
Personalization	
Timing: on-demand support and 24/7 availability *“*Of course, you have people around you who support you when you’re trying to quit smoking. But Sam was there 24/7. So even late at night or early in the morning, I could reach out to Sam.” (M3, abstinent and increased PA at T1)	—
Content: personalized step goals Interviewer: *“*Which aspects of the program did you like?*”* P: *“*Definitely the step goal. You want to reach it, so that’s motivating when you get it. I used it a lot.*”* (H4, abstinent and increased PA at T1)	—
Content: variety in short optional activities *“*What I liked about the program was the variety – you didn’t keep getting the same thing, but you could choose. I enjoyed doing the relaxation exercises and watching the information videos.” (H3, abstinent and increased PA at T1)	Content: insufficient variety in short optional activities *“*I found them a bit underwhelming [referring to the PF activities], because they didn’t really add anything for me. […] They didn’t shift my mindset. […] You know, you’ve been at it for so long, and you already know everything so well – that smoking is bad for you. You’ve already tried quitting a thousand times.” (M2, smoking and increased PA at T1) *“*Maybe expand the activities. […] For example, once you’ve completed an activity, you could move on to a next step – like, ‘you’ve finished this level, now you can continue to the next level’ or something like that.” (H1, abstinent and decreased PA at T1)
—	Content: insufficient variety during execution phase *“*The week after, I just got exactly the same questions again, and I would have preferred if the tone or the way the questions were asked had been a bit different. So that you’d feel like you were progressing further in the program together with Sam.” (M3, abstinent and increased PA at T1)
Technical aspects and usability	
Clear and easy to use *“*It wasn’t disappointing. I mean, I’m obviously not 20 anymore, so I didn’t grow up with apps and computers. I don’t think I’m completely incapable, but it’s not my hobby. So it’s always a bit nerve-wracking, but it’s quite manageable, even if you’re less skilled.*”* (H3, abstinent and increased PA at T1)	Complicated installation and app integration *“*The installation was technically challenging. It was difficult that you needed to link the Garmin Connect app with the NiceDay app and also to add Sammy as a chatbot. That was a bit complicated and tricky, and it should be simpler.” (L1, smoking and increased PA at T1)
—	Technical issues *“*Except when it malfunctioned, I thought: here we go again. ‘I haven’t heard anything for a while,’ Sam said. Then I thought, well, it’s not that long, Sam, but yeah, you can’t really express that (laughing).” (H3, abstinent and increased PA at T1)
—	Smartwatch connection issues *“*I contacted you [the researchers] again at some point because the step counter wasn’t working. I couldn’t get it to work. And eventually, I sort of gave up – like, ‘Never mind, I don’t want to be a bother.’ If I’d had a mid-program evaluation talk [with a researcher], I might have brought it up again. I would’ve really appreciated that.*”* (M5, abstinent and increased PA at T1)
Responsive technical support and troubleshooting *“*And you [the researchers] were always very accessible, which makes a difference. If something doesn’t run smoothly, it’s easy to get in touch. […] It’s just a quick email and you get an immediate reply, so that makes things less frustrating when something goes wrong.” (H1, abstinent and decreased PA at T1) *“*A few times, I saw that zero steps were recorded, and I wondered how that could be. Then I checked the booklet [the Perfect Fit paper manual]: ‘Oh right, every time something happens or if you put your phone in battery-saving mode, you have to reopen the app*.’”* (H2, abstinent and decreased PA at T1)	—

a Interview ID is based on Perfect Fit usage: low (L), moderate users (M), and high (H) users.

b PA: physical activity.

c T1: postintervention.

d Not applicable.

e Quotes placed opposite each other illustrate opposing experiences reported by different participants; not every strength has a corresponding point for improvement, and not every point for improvement corresponds to a strength.

None of the 12 interview participants reported feeling uncomfortable or unsafe when sharing personal information during the intervention. Some noted that the questions were not particularly sensitive and/or attributed their sense of safety to the study’s affiliation with Leiden University Medical Center, an academic hospital in the Netherlands:

*Yes, I felt safe sharing information. Although I’m not exactly sure why – maybe because it was still in the research phase and the data was handled confidentially for a good cause. […] And it wasn’t that much personal information anyway.*

[L3, smoking and increased PA at T1]

### Acceptance of the Virtual Coach

**Figure 2.**
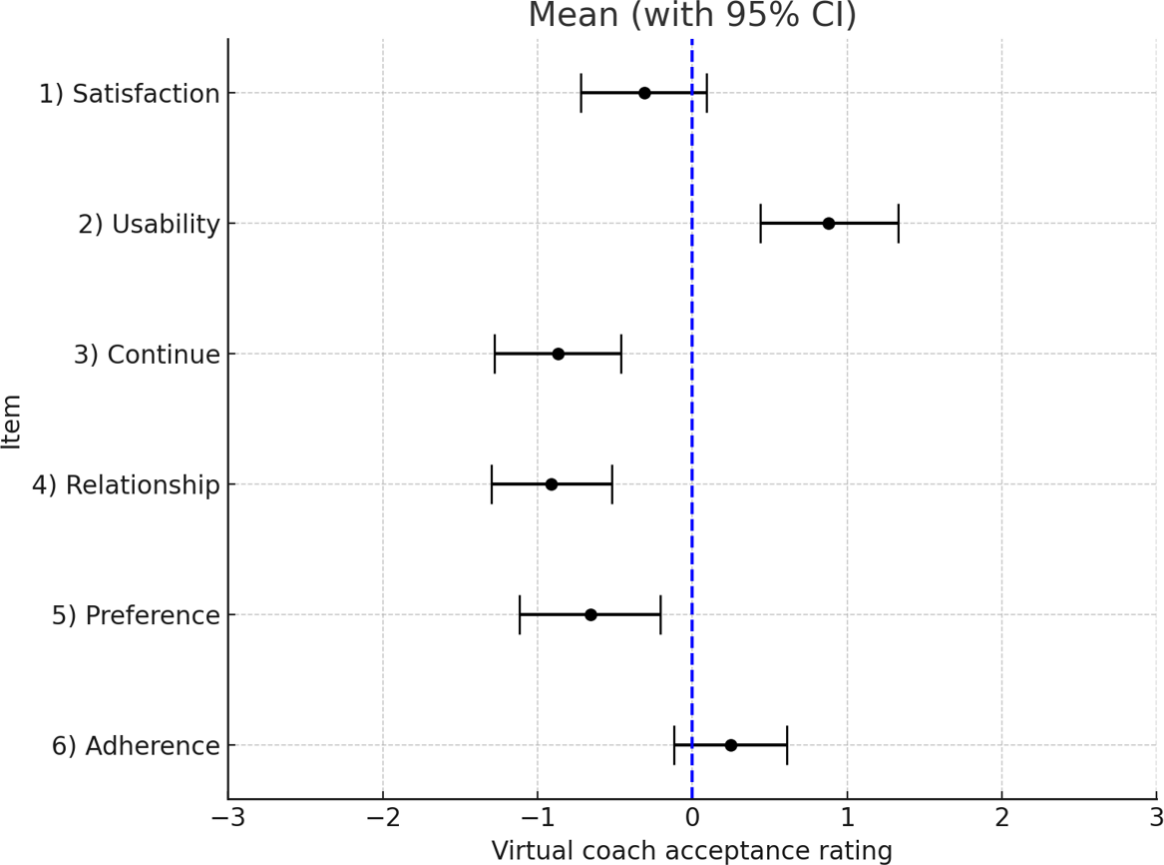
Mean scores (95% CIs) for the 6 virtual coach acceptance items (with varying response labels, eg, difficult-easy), with higher scores indicating more positive attitudes toward the virtual coach.

At postintervention, the mean virtual coach’s acceptance rating was −0.27 (SD 1.30; n=77) on a scale from −3 to 3. Figure 2 shows the mean scores and 95% CIs for each of the 6 items assessing coach acceptance. The item on usability received the highest rating, whereas the coach-user relationship was rated the lowest.

Qualitative findings provided context and depth to the quantitative results, for instance, regarding the moderately negative appraisal of the virtual coach, *“*I think the setup [of PF] is really great. […] But the virtual coach – yeah, I actually liked that the least.” (L1, smoking and increased PA at T1). Participants frequently described the coach’s communication as repetitive and static in style and content. Many expressed a desire for more flexibility, suggesting the coach should be able to adapt to personal situations (eg, being ill and unable to exercise) or user input (eg, by reflecting on user messages or tailoring content accordingly).

*Probably because he [the virtual coach] didn’t learn from my experiences. […] At one point, there was an exercise where you had to note why things were going well or not. I typed it in my notes or the app, but it wasn’t read or analyzed. […] So that doesn’t help. Maybe it’s useful for a bit of self-reflection, but […] that coach didn’t learn anything from me.*

[L3, smoking and increased PA at T1]

Still, several participants expressed appreciation for the support provided by the coach and its friendly, nonjudgmental, and empathetic tone:

*Not judgmental and really encouraging when you’d done something well. Like occasionally checking in: ‘How are you feeling?’, ‘How’s it going?’, ‘ Any difficult moments?’, ‘No?’, ‘ Great job.’*

[H1, abstinent and decreased PA at T1]

Participants held differing views on specific features of the coach. For example, the frequency of notifications and the chatbot format were appreciated by some but criticized by others. The fact that Sam was a chatbot, rather than a human coach, was seen as a benefit by some participants, as it offered a sense of anonymity, nonjudgment, and autonomy. In contrast, others perceived it less favorably, noting that it reduced their sense of personal accountability and placed full responsibility on themselves:

*Of course it’s a computer, but I actually liked that. […] Constantly talking to a real person – ‘Why did you smoke?’ – that can get frustrating. Like: I don’t know, just drop it. And with this program, you could choose what you wanted and how you wanted to receive support.*

[H3, abstinent and increased PA at T1]

*You know, a lot of things in the app are quite noncommittal, and I wouldn’t mind a stricter Sam. […] Less ‘you may’, and more ‘you must’.*

[M1, smoking and increased PA at T1]

These different experiences of participants appeared to influence their perceived bond with coach Sam. While the above-mentioned positive aspects seemed to contribute to a sense of support, several participants felt that Sam being a computer made it difficult—if not impossible—to form a real connection, unlike with a human coach. At the same time, participants noted that developing a human-like bond was not necessary to find Sam’s support helpful or to have a positive experience with PF, *“*What did I think of coach Sam? […] A nice computer, right? (laughs)” (H2, abstinent and decreased PA at T1).

### Exploratory Analyses

#### Sensor Data Issues

Given the unexpectedly frequent issues with the connection between the smartwatch and the virtual coaching system (see Methods, Outcomes section, “Sensor data issues”), the extent and nature of these issues were explored post hoc.

Among the 87 participants with available PF usage data, the median percentage of days with available step count data during the intervention period (ie, from first to last activity in the PF app) was only 43.1% (range 0.0%‐98.3%), indicating substantial missing data. Several participants mentioned smartwatch connection issues during contact with the research team. These issues occurred despite participants wearing the smartwatch, suggesting that the missing data were primarily due to technical issues. Supporting this, exploratory analyses showed that the missing step count data were not associated with specific days of the week (*χ^2^*_6_=5.97; *P*=.43; Cramér *V*=0.03), suggesting that missing data were not due to patterned nonwear (eg, on weekends) but more likely due to technical issues. This is further supported by the significant association between operating system and the percentage of days with step count data (*U*=670.00; *Z*=−2.14; *P*=.03; *r*=−0.23). Specifically, Android users (n=51, 58.6%; median rank=48.86) had significantly more days with data than iPhone users (n=36, 41.4%; median rank=37.11), possibly reflecting connectivity issues specific to the operating system. In addition, there were significant differences between PF usage groups in the percentage of days with step count data (*H*_2_=24.29; *P*<.001). Post hoc comparisons showed that participants in the high-usage group (median 85.6%, range 0.0%‐98.3%) had significantly more days with step count data than those in the moderate-usage (median 26.8%, range 0.0%‐88.8%; *P*<.001) and low-usage groups (median 30.8%, range 0.0%‐97.8%; *P*=.003). These findings could indicate that persistent connection issues might have contributed to lower usage. This interpretation is supported by qualitative data: some participants reported feeling demotivated or even discontinuing their use of PF due to unresolved connection issues between the smartwatch and coach (also see the quotation accompanying the point for improvement “Smartwatch connection issues” in Table 2):

*I sent several emails about it, but it just kept being difficult that Sam didn’t register my steps. And that was actually demotivating – when Sam would say, ‘you’ve walked zero steps today.’ Then I’d try again [to fix it], and sometimes it worked, sometimes it didn’t. I still can’t put my finger on it. But yes, that did demotivate me at times.*

[M3, abstinent and increased PA at T1]

Although potential causes of the sensor connection issues were identified, many remained difficult to trace. Troubleshooting strategies had limited and often temporary effects. Technical lessons learned and recommendations for future research are provided in Multimedia Appendix 3.

#### Associations

To explore associations between baseline characteristics and PF usage, satisfaction, usability, and coach acceptance, we conducted exploratory quantitative analyses, complemented by qualitative data that provided contextual insight. As PF was specifically designed to be suitable for individuals with lower SEP and eHealth literacy, results related to these variables and significant associations with other variables (*P*<.05) are highlighted here. Full details and additional nonsignificant results are provided in Table S3 in Multimedia Appendix 2.

Age differed significantly between PF usage groups. Post hoc comparisons showed that participants in the high-usage group (mean 58.39, SD 12.79) were older than those in the low-usage (mean 50.30, SD 13.95; *P*=.05) and moderate-usage groups (mean 48.47, SD 13.85; *P*=.01). This contrasts with some of the interview data, in which older participants expressed that they expected PF to be easier to use for younger individuals, often linking this to digital skills:

*When I typed something and Sam replied, it happened very quickly, and every time I had to scroll back to read it. But as soon as he typed something new, it jumped up again. I found that really annoying. […] Younger people probably find that easier, they’re also faster than me.*

[M4, 61 years]

Digital literacy was also mentioned more generally as influencing ease of use, particularly in dealing with errors, such as the smartwatch connection issues:

*You had to first open and then close the app again to get a connection. Luckily, I’m quite tech-savvy, so I sorted it out quickly. But I can imagine that being very frustrating if you’re not.*

[M1, 48 years]

However, a few older participants with lower digital skills noted that they still found PF manageable (see quotation accompanying the strength “Clear and easy to use” in Table 2).

In addition to age, quantitative analyses showed that the eHealth literacy scale 4—*“*Feel safe and in control”*—*was weakly, positively correlated with the perceived usability of PF. This suggests that participants who felt more ownership over their data and perceived it as secure reported higher usability of PF.

No significant associations were found in the quantitative analyses between SEP, eHealth literacy subscales (except for scale 4), and any of the primary outcomes. This suggests that PF may have been perceived as similarly feasible and acceptable across these groups. Interestingly, a trend-level difference in satisfaction with PF was observed across SEP groups, with somewhat higher satisfaction among participants with low SEP (mean 3.03, SD 0.67) compared to middle (mean 2.90, SD 0.70) and high SEP participants (mean 2.56, SD 0.71). However, a post hoc power analysis using the largest observed effect size for SEP and eHealth literacy associations (*f*=0.30) indicated only about 61% power with the current sample size. This likely limits the ability to detect significant differences.

### Secondary Outcomes

#### Recruitment, Response, Consent Rates, and Recruitment Strategies

Recruitment took place over approximately 6 months. In total, 361 individuals expressed interest in the study, of whom 222 were screened for eligibility, resulting in a response rate of 0.61. The consent rate among those eligible was 0.83 (see Figure 1). These rates indicate both the feasibility of the recruitment strategies and substantial initial interest in PF. The target sample size of 100 was reached before all eligible individuals could be enrolled, further suggesting promising user interest in a virtual coach-based mHealth intervention for smoking cessation and PA promotion.

Among the 100 participants enrolled, recruitment was most effective through newsletters from 2 health insurance companies (n=37, 37.0%), followed by social media (mainly Facebook advertisements) and recruitment via family, friends, or work contacts (both n=21, 21.0%). More labor-intensive strategies, such as in-person flyer distribution or a local newspaper interview, yielded only a few participants (both n=4; see Table S4 in Multimedia Appendix 2).

Since recruiting participants from lower SEP backgrounds is often reported as challenging [14], we also examined recruitment effectiveness across SEP groups. No significant differences in recruitment strategy were found between SEP groups (*χ^2^*_18_=20.06; *P*=.33; Cramér *V*=0.33).

#### Study Adherence

A significant association was found between PF usage (low, moderate, and high) and study adherence at T1 (*χ^2^*_2_=9.32; *P*=.009; Cramér *V*=0.31). T1 completers (n=77) were more likely to be in the high-usage group (n=26, 33.8%) than noncompleters (n=7, 9.1%). Conversely, most noncompleters (n=15, 63.6%) were in the low-usage group, compared to 29.9% (n=23) of completers.

## Discussion

### Principal Findings

This single-arm, pre-post, convergent mixed methods study primarily aimed to investigate the feasibility and acceptability of PF, an mHealth intervention with a virtual coach offering real-time, personalized feedback to support both smoking cessation and PA promotion. Additional aims included exploring associations between baseline characteristics and feasibility and acceptability outcomes, as well as examining the feasibility of conducting the research study. Overall, PF showed adequate feasibility and acceptability, and study procedures proved feasible. Furthermore, the mixed methods design yielded valuable insights into participants’ experiences and areas for improving PF and virtual coach interventions in general.

The main findings showed that PF usage varied considerably across participants. Descriptive analyses indicated moderate-to-good satisfaction and usability of PF, but virtual coach acceptance was somewhat negative. High PF users reported greater satisfaction with PF, usability of PF, and coach acceptance. Frequent connection issues between the smartwatch and coach emerged as a disruptive factor, reported more often in the low- and moderate-usage groups than in the high-usage group. Qualitative results provided in-depth insights into PF’s feasibility and acceptability, encompassing both positive and negative experiences. Exploratory analyses suggested that high PF users were older than those with moderate and low usage and that the eHealth literacy scale “Feel safe and in control” was weakly positively correlated with usability. Importantly, PF was perceived as similarly feasible and acceptable across SEP groups, aligning with one of the research project’s goals. Finally, there was substantial initial interest in PF, and study retention postintervention was 77%, which is relatively high given typical attrition in eHealth studies [21,71].

### Understanding Variation in Usage and Experiences

Variation in PF usage and experiences may first be explained by user engagement, conceptualized as (1) the extent of usage and (2) the subjective experience (ie, attention, interest, and affect) [72]. Regarding usage extent, quantitative data showed that weekly coach interaction and intervention duration were generally high; yet, completion of core intervention components varied widely. This may reflect the intervention’s personalization but also differences in satisfaction and usability. Subjective engagement, inferred from qualitative data, also varied: some participants found PF motivating and enjoyable, whereas others did not. Increased attention and interest were reported when content prompted reflection or self-reflection, felt personally relevant, or was delivered empathetically. Participants reported the need for evolving goals and content to maintain relevance, challenge, and a sense of progress. This may support perceived competence, potentially leading to high engagement or “flow,” consistent with psychological flow theory [73]. This emphasizes the value of personalization, as shown in prior studies [32,74,75]. A second explanation for variation in usage and experiences concerns technical issues. These issues caused frustration and loss of interest for some participants, mirroring findings from a previous review [25]. A particularly disruptive issue was the smartwatch-coach connection, interfering with step tracking and goal personalization. Findings indicated that these issues were associated with lower PF usage and early dropouts, consistent with a review on mHealth interventions for PA promotion [75]. Although many connection issues were difficult to trace, we shared lessons learned (Multimedia Appendix 3) to help mitigate them in future studies. For example, implementing real-time monitoring systems may help detect missing sensor data early and allow researchers to intervene before data loss undermines engagement or intervention delivery. Together, these findings highlight 2 potential factors underlying variability in usage and experiences, offering guidance for future interventions.

### Challenges and Opportunities for the Virtual Coach

The virtual coach was the least appreciated component of PF. Qualitative feedback showed participants generally valued its supportive, empathetic, and nonjudgmental style but were critical of its repetitive, static communication and limited adaptability to user input or personal situations. Similar concerns have also been reported in a previous review of virtual coaches for smoking cessation [25]. One potential strategy to improve the coach’s communication is the use of natural language processing, which can increase variation and adaptability by training the system to classify and generate responses based on large textual datasets [1,21,25,76]. While recent advances make this a promising approach that might increase the coach’s acceptability, such models can produce potentially misleading, inappropriate, or nonfactual outputs [21,77,78]. It remains crucial to balance flexibility (eg, responsiveness to context) with controllability, consistency in content delivery, and safety. As rule-based systems offer greater control [21,76], a hybrid approach combining rule-based and probabilistic techniques may be most suitable for future virtual coach interventions [79].

### Accessibility and Inclusivity

We aimed to make PF accessible to individuals often underserved by health behavior change interventions, including those with a lower SEP, limited eHealth literacy, or digital skills [21,25]. Exploratory analyses revealed no significant differences in feasibility and acceptability across SEP groups. Although the study was underpowered to detect small effects, there was a trend toward higher PF satisfaction among participants with a lower SEP. Moreover, participants scoring higher on the “feel safe and in control” eHealth Literacy subscale reported greater usability of PF. Qualitative feedback, especially from older participants with self-reported lower digital skills, indicated that they expected PF to be easier for younger participants or would have appreciated additional support (eg, from a human assistant). However, experiences varied, as some older participants with limited digital skills found PF manageable. Interestingly, participants in the high-usage group were significantly older than those in the low- and moderate-usage groups. These findings suggest that older age does not necessarily limit the ability to use PF, possibly because PF was designed to be suitable for individuals with lower digital skills. Furthermore, older users may have had fewer prior experiences with digital interventions, which can lead to lower expectations and, consequently, a more positive appraisal of the intervention [75]. Finally, dropouts did not differ significantly from completers in SEP, age, or eHealth literacy. These findings are noteworthy given concerns that virtual coaches (for smoking cessation) may exacerbate health disparities [21,25]. Developing accessible and inclusive interventions is therefore essential, and these findings indicate that PF may be a promising step in that direction.

### Strengths and Limitations

This study has several strengths. First, PF integrates behavior change techniques commonly used in smoking cessation [37] and PA promotion [38], as well as behavioral theories. Additionally, we incorporated features previously reported as lacking in virtual coaches, such as relational strategies [25], and addressed literature gaps, including transparent intervention reporting [25], which we provided in our published protocol [43]. Moreover, PF was developed by an interdisciplinary team in collaboration with end users [36], likely enhancing feasibility and acceptability. Second, we conducted an in-depth evaluation of PF’s feasibility and acceptability. These aspects remain underresearched in virtual coaches [1], despite their importance for long-term adoption [24-26,33,34]. Our convergent mixed methods approach captured both general patterns and nuanced user experiences, helping to identify key strengths and areas for improvement. Third, initial interest in PF was high, and the retention rate was approximately 77% at postintervention (4 months after baseline). This is a positive outcome given the typically high dropout rates in digital interventions [21,71]. Furthermore, recruiting participants from lower socioeconomic backgrounds is often challenging [14] but essential to promote digital inclusion. Therefore, we applied recruitment strategies to reach a broad, diverse audience, resulting in a SEP distribution comparable to the general Dutch population [68].

This study also has some limitations. First, we did not include a dedicated user engagement questionnaire (eg, the short form of the User Engagement Scale [80]), which might have provided a more explicit assessment of engagement. However, there is ongoing debate about the best way to measure engagement in virtual coach interventions, and many studies rely solely on objective usage data, overlooking users’ subjective experiences [1,25]. By applying a mixed methods approach, we inferred subjective engagement from qualitative data and identified potential barriers and facilitators. Second, an error in the SEP measurement prevented distinguishing between lower-level (1-2) and middle-level (3-4) vocational education. As a result, all participants with vocational education were classified as middle SEP, likely underestimating the lower SEP group. Third, postintervention completers generally showed higher PF usage, which may have led to an overestimation of feasibility and acceptability. Furthermore, although we aimed to recruit a diverse group for the interviews, participants who were more engaged with PF may have been more likely to participate. To address this, we explicitly invited participants with different levels of PF usage, leading to interviews with 3 low PF users. This may have helped mitigate potential overestimation.

### Research and Practical Implications

Several implications emerged for further development of PF and virtual coaches in general. Key areas for improvement included greater content variation, enhanced adaptability of the coach to user input and personal situations, integration of intervention into a single app, and a more reliable smartwatch-coach connection. Although PF incorporated personalization strategies (eg, adaptive step goals), qualitative feedback highlighted the need for additional personalization. For example, adapting the coach to users’ preferred coaching style (eg, more directive vs autonomy-supportive) may enhance engagement [81,82]. As engagement can change over time [28], personalization based on dynamic user states (eg, motivation) may also be beneficial [25,83]. Besides enhancing personalization, identifying which populations these interventions best serve is important. Our findings revealed varying preferences; for instance, some participants valued a virtual coach for anonymity, low-threshold access, and a sense of control, while others favored a human coach for greater accountability. These insights can guide future research on aligning population characteristics with different delivery modes of virtual coach interventions. At the same time, broad accessibility should be promoted to avoid exacerbating health disparities, for instance, by exploring different implementation formats. PF was evaluated as a standalone intervention, which may be suitable for those preferring self-guided support or facing barriers to human care. Adding low-level human involvement could further support individuals with lower digital skills (as indicated by our qualitative findings) or lower SEP [14], while considering limited health care resources and ethical allocation. Strategies such as human feedback messages [83] or adaptive designs escalating from low-intensity digital support to human involvement at certain moments [84] may help achieve this balance. Future research should explore how to balance effectiveness, resource use, and equity in virtual coach interventions.

### Conclusions

PF shows adequate feasibility and acceptability as a virtual coach-based intervention for smoking cessation and PA promotion. It is accessible to groups often underserved by behavior change interventions, such as those with lower SEP or older age. Usage varied considerably, potentially due to differences in engagement and technical issues (eg, smartwatch-coach connection). Participants valued the coach’s empathetic style but noted limitations in its adaptability. This underscores the need for additional personalization, for example, through hybrid approaches combining rule-based systems with natural language processing. Variation in participant experiences highlights the importance of identifying which populations are best served by virtual coaches while promoting broad accessibility to avoid exacerbating health disparities. One strategy is to add low-level human involvement while accounting for limited health care resources.

Overall, PF shows potential as an accessible, inclusive multibehavior change intervention that could benefit public health. This study lays the groundwork for follow-up research evaluating PF’s effectiveness and suitable implementation strategies, and provides recommendations for the development of future virtual coach interventions.

## Supplementary material

10.2196/83456 Multimedia Appendix 1 Detailed reporting of patient and public involvement (PPI) using the Guidance for Reporting Involvement of Patients and the Public (GRIPP) 2 short form.

10.2196/83456 Multimedia Appendix 2 Data preparation and additional results.

10.2196/83456 Multimedia Appendix 3 Sensor connection issues: technical lessons learned and recommendations.

10.2196/83456 Checklist 1 Good Reporting of a Mixed Methods Study (GRAMMS) checklist.

## Abbreviations

eHLQ: eHealth Literacy Questionnaire
FTND: Fagerström Test for Nicotine Dependence
GRAMMS: Good Reporting of a Mixed Methods Study
GRIPP2: revised version of Guidance for Reporting Involvement of Patients and the Public
GSLTPAQ: Godin-Shephard Leisure-Time Physical Activity Questionnaire
HSD: honestly significant difference
mHealth: mobile health
PA: physical activity
PF: Perfect Fit
PPI: patient and public involvement
SEP: socioeconomic position
SMART: specific, measurable, achievable, relevant, and time-bound
SUS: System Usability Scale
WHO: World Health Organization
