# Supplementary material for: Evaluation of the Feasibility and Acceptability of Perfect Fit, a Virtual Coach–Based mHealth Intervention for Smoking Cessation and Physical Activity in Adults: Mixed Methods Study
Source: JMIR Hum Factors. 2026 Jul 14;13:e83456. doi: 10.2196/83456 (PMC13367948; doi:10.2196/83456)
Supplement: Multimedia Appendix 1 [file humanfactors-v13-e83456-s001.docx]

**Appendix 1.** *Detailed reporting of patient and public involvement (PPI) using the Guidance for Reporting Involvement of Patients and the Public (GRIPP) 2 short form*

| **Table S1**  *PPI in developing Perfect Fit: Reporting according to the GRIPP2-SF [1].* | |
| --- | --- |
| **Section and topic GRIPP2-SF** | **Description** |
| *1: Aim*  Report the aim. | The aim of PPI in this project was to enhance the relevance, usability, accessibility, and effectiveness of the Perfect Fit (PF) intervention, as well as to improve certain study procedures. Potential end-users, experts on relevant topics, and professionals likely to work with PF were involved to incorporate their perspectives, ideas, and experiences. By engaging end-users, the research team also aimed to empathize with the target population’s perceptions and align the intervention and study procedures with their needs, preferences, and skills. Furthermore, one of the aims of the PF project was to make the intervention accessible and relevant for individuals with a lower socioeconomic position, limited eHealth literacy, or limited digital skills. Therefore, representatives of this population were involved in various ways throughout the project. |
| *2: Methods*  Provide a clear description of the methods used for PPI in the study. | An advisory panel of potential end-users with experience or intent to quit smoking was established one year into the project. The panel consisted of three active members, with diverse characteristics, such as being over 65 years old, having little or no prior research experience, limited digital skills, or a lower socioeconomic position. The panel was involved throughout the five-year PF project, providing input across different research phases and multiple studies, with a particular focus on the development of the PF intervention.  In addition to recurring involvement of the advisory panel, one-time PPI activities were conducted with other end-users to gather further feedback on specific intervention components or research questions. For instance, focus groups were held with individuals aged 45 and older, with a low-to-middle socioeconomic position and insufficient physical activity levels, to discuss potential features of PF.  Finally, experts (e.g., lifestyle coaches, psychologists), who may work with PF in the future, were consulted through interviews to incorporate their professional perspectives into the intervention.  Further details on PPI activities can be found in the published article on the development of PF [2]. |
| *3: Study Results*  Outcomes—Report the results of PPI in the study, including both positive and negative outcomes. | PPI contributed to the development of PF and the current study in several ways:   - End-users provided input on the **user journey** of the intervention, which informed the overall structure of PF. They highlighted the importance of **tailoring** the timing, content, and frequency of engagement with intervention components, leading to more personalized design features (e.g., the option to adjust the duration of the preparation phase). - End-users contributed to the **content** of PF. For example, by selecting and suggesting relevant optional short activities and providing practical examples based on their own experiences with quitting smoking or becoming more physically active (e.g., peeling a mandarin or drinking water as an alternative to smoking). - End-users collaborated on the **design** of PF, for example, by thinking along on the name and profile picture of the virtual coach. - Researchers from the research team visited a local community center that one of the advisory panel members frequently visits. There, they spoke with individuals who smoke to gain insights into their daily experiences. - End-users reviewed virtual coach dialogs, which improved the **readability** of the dialogs and helped to ensure the **tone** of the coach was appropriate and user-friendly. - End-users assisted in **participant recruitment** for the study. - End-users supported the **interpretation of findings** from the current study. - Experts on the development of accessible digital interventions provided feedback on how to improve the **accessibility** of PF, particularly for individuals with lower eHealth literacy or digital skills. - Interviews with experts (e.g., lifestyle coaches) provided an overview of commonly effective techniques (e.g., goal-setting, social support) when coaching individuals to quit smoking and increase physical activity. They also provided recommendations and cautions that informed the final mHealth intervention. |
| *4: Discussion and conclusions*  Outcomes—Comment on the extent to which PPI influenced the study overall. Describe positive and negative effects. | Overall, the PPI activities were perceived as a valuable addition to the development of PF and the research study. Input from end-users helped shape the structure and content of PF, contributing to its personalization, user-friendliness, and acceptability. It also informed practical aspects of the study procedures, such as recruitment strategies. The integration of perspectives, examples, and ideas from end-users and experts likely increased the accessibility and relevance of PF.  Several factors contributed to the positive impact of PPI in this project. Various PPI activities were used, ranging from long-term advisory panel engagement to one-time end-user and expert involvement, allowing for diverse and complementary insights. The ongoing collaboration with the advisory panel enabled the research team to rapidly incorporate feedback and test intermediate versions of the intervention, which was very valuable in the iterative and fast-paced development process of digital interventions. This iterative process supported a more responsive development cycle. Furthermore, the collaboration helped researchers better understand the lived experiences and needs of the target population, which likely contributed to better alignment of the intervention with this population. End-of-project evaluations with the advisory panel indicated that members felt they contributed to the project and that their input was valued. This mutual appreciation likely strengthened the collaborative process.  There were also some limitations. Since this was the first time the research team engaged in such intensive and diverse forms of PPI, it sometimes took time to determine the most appropriate approaches. In addition, the advisory panel was involved early in the project, but after the funding proposal had already been developed. Even earlier involvement might have been valuable in shaping some of the initial design choices. |
| *5: Reflections/critical perspective*  Comment critically on the study, reflecting on the things that went well and those that did not, so others can learn from this experience. | While the PPI approach contributed positively to PF and was generally well-received by both the research team and advisory panel, some challenges and lessons learned for future projects emerged. The research team was new to such an intensive level of PPI, which meant some trial and error in planning and organizing PPI activities. In hindsight, involving the advisory panel even earlier in the project and developing a more concrete PPI plan from the start would likely have facilitated more efficient planning and allocation of time. Additionally, engaging underrepresented end-users, such as individuals with lower socioeconomic position or digital skills, often requires more time-intensive and tailored strategies (e.g., trust-building, visiting community centers, or conducting individual sessions to assess intervention accessibility and readability). Future projects might benefit from allocating additional resources specifically for these activities and from building in flexibility to adapt the PPI process as needed. This would support a more inclusive and responsive approach to PPI.  On the positive side, the collaborative process fostered valuable learning for both researchers and involved end-users and enhanced the quality and relevance of PF and the study. |

**References**

1. Staniszewska S, Brett J, Simera I, Seers K, Mockford C, Goodlad S, et al. GRIPP2 reporting checklists: tools to improve reporting of patient and public involvement in research. Res Involv Engagem. 2017;3:13. PMID: 29062538. doi: 10.1186/s40900-017-0062-2.

2. Versluis A, Penfornis KM, van der Burg SA, Scheltinga BL, van Vliet MH, Albers N, et al. Targeting key risk factors for cardiovascular disease in at-risk individuals: developing a digital, personalized, and real-time intervention to facilitate smoking cessation and physical activity. JMIR Cardio. 2024;8(1):e47730. PMID: 39705698. doi: 10.2196/47730.
