# Supplementary material for: Evaluation of the Feasibility and Acceptability of Perfect Fit, a Virtual Coach–Based mHealth Intervention for Smoking Cessation and Physical Activity in Adults: Mixed Methods Study
Source: JMIR Hum Factors. 2026 Jul 14;13:e83456. doi: 10.2196/83456 (PMC13367948; doi:10.2196/83456)
Supplement: Multimedia Appendix 2 [file humanfactors-v13-e83456-s002.docx]

*Data preparation and additional results*

**Data preparation**

Age was calculated based on participants’ birth year. SEP was derived from participants' highest completed educational level [1]. Due to a measurement error in vocational education, it was not possible to differentiate between lower-level (1-2) and middle-level (3-4) vocational education. As a result, all participants with vocational education were classified as middle SEP, instead of partly lower and partly middle SEP, as originally intended. This resulted in the following SEP categories: low (no education, primary school, and pre-vocational secondary education), middle (higher-level, pre-university secondary education, and all vocational education), and high (higher professional and university education). Because of skewed baseline PA level data (GSLTPAQ) and extreme outliers, values above the 95th percentile (cut-off = 79) were winsorized [2], meaning that they were replaced by the value at the 95th percentile to reduce the influence of extreme scores [2]. The 95^th^ percentile was chosen over the 99th percentile because it produced a more stable and representative central tendency.

To avoid small cell sizes when exploring associations between baseline and outcome variables (secondary study aim), several variables were dichotomized: gender (female vs. male), presence of a physical or mental condition (none vs. one or more), and intention to quit smoking and to become sufficiently physically active (within 1 month vs. not within 1 month but sometime in the future). PF usage was categorized into three groups: low usage (0-7 core components completed, corresponding to dropout during the preparation phase), moderate usage (8-14 core components completed, corresponding to dropout between the start of the execution phase and the first half of this phase), and high usage (15-21 core components completed, corresponding to dropout between the second half of the execution phase and the end of the intervention).

For the exploratory sensor data analyses, days with step counts <1000 were marked as missing, assuming the smartwatch was either not worn or failed to connect properly with the coach. This threshold was based on prior research [3, 4]. Furthermore, sensitivity analyses comparing <100, <300, and <1000 cut-offs showed that the <1000 threshold was a conservative and reliable choice, increasing missing values by only 1.4%.

Outliers and statistical assumptions were checked, and scale reliability was calculated where appropriate.

**Qualitative interview sample characteristics**

| **Table S1**  *Baseline characteristics Interview sample (n = 12)* | | | | | |  |
| --- | --- | --- | --- | --- | --- | --- |
| **Interview ID^a^** | **Gender** | **Age in years** | **Socioeconomic position** | **eHLQ scale 1 to 5, median (range)** | **Perfect Fit usage** |  |
| **L1** | Male | 36 | High | 3.00 (2.60-3.40) | Low |  |
| **L2** | Male | 54 | Middle | 3.00 (2.60-3.00) | Low |  |
| **L3** | Male | 68 | Middle | 3.80 (3.00-4.00) | Low |  |
| **M1** | Male | 48 | Middle | 2.60 (2.40-3.40) | Moderate |  |
| **M2** | Male | 54 | Low | 3.20 (3.20-4.00) | Moderate |  |
| **M3** | Female | 56 | Middle | 2.80 (2.20-3.20) | Moderate |  |
| **M4** | Female | 61 | Middle | 3.20 (3.00-3.80) | Moderate |  |
| **M5** | Female | 62 | High | 3.80 (3.40-4.00) | Moderate |  |
| **H1** | Female | 35 | Low | 3.00 (2.60-3.00) | High |  |
| **H2** | Male | 44 | Middle | 2.60 (2.20-2.80) | High |  |
| **H3** | Female | 62 | Middle | 2.80 (2.40-3.20) | High |  |
| **H4** | Male | 77 | High | 3.00 (2.80-3.00) | High |  |
| *Note.*  eHLQ = eHealth Literacy Questionnaire.  ^a^Interview ID is based on low PF users (= L), moderate PF users (= M), and high PF users (= H). | | | | | | |

**Additional results: Feasibility and acceptability of PF**

***Frequency contact virtual coach***

At post-intervention (T1), participants were asked to indicate how many times per week they had interacted with the virtual coach during the PF intervention. Among the 77 participants who completed T1, the median reported frequency of contact was 3 times per week (range = 0-34). This aligns with the expected contact frequency, as PF included 21 core components spread across an expected intervention duration of 16 weeks. Additionally, participants could initiate optional activities and dialogs at any time (e.g., 25 short optional activities, or the high-risk situation and (re)lapse dialog in case of difficult moments).

***PF usage: Completed core components and optional short activities***

Table C2 provides a general overview of the number of completed core intervention components and optional short activities, stratified by PF usage category (low, moderate, high). Categories were defined based on the total number of completed coach-initiated core components. It should be noted that participants did not always complete every consecutive component; for example, some participants skipped preparation or execution phase components but still completed the final closing dialog. Although these components were recommended by the virtual coach, if a participant did not respond within a set time, the system automatically advanced to the next component.

| **Table S2**  *Completed core components and optional short activities, stratified by PF usage category (n = 87).* | | | |
| --- | --- | --- | --- |
| **Perfect Fit usage category** | **Number of completed core components** | ***n*** | **Number of completed optional short activities, median (range)** |
| Low | 0-7 | 25 | 1.00 (0.00-15.00) |
| Moderate | 8-14 | 34 | 3.50 (0.00-16.00) |
| High | 15-21 | 28 | 9.00 (1.00-46.00) |

**Additional results: Associations**

To address the secondary study aim, exploratory analyses were conducted to examine associations between baseline variables and the primary outcomes: PF usage, satisfaction with PF, usability of PF, and acceptance of the virtual coach. Depending on the level of measurement and distributional assumptions, one-way ANOVAs, Kruskal-Wallis tests, chi-square tests, and Pearson’s and Spearman’s correlations were performed (see Table C3).

| **Table S3**  *Overview of exploratory association analyses between baseline characteristics and primary outcomes.* | | | | | |
| --- | --- | --- | --- | --- | --- |
| **Outcome measure** | **Baseline variable** | ***n*** | **Test statistic (df)** | **P-value** | **Effect size** |
| **PF usage:**  **low, moderate, high** | Age | 99 | *F*(2,96) = 4.53 | .013* | *η^2^* = .09 |
|  | Gender: Female/male | 98 | *χ^2^*(2) = 1.49 | .48 | Cramér's *V* = .12 |
|  | Socioeconomic position:  low, middle, high | 98 | *χ^2^*(4) = 2.40 | .66 | Cramér's *V* = .11 |
|  | Physical or mental (chronic) condition(s):  Yes/no | 99 | *χ^2^*(2) = 0.06 | .97 | Cramér's *V* = .03 |
|  | eHLQ 1: Using technology to process health information | 99 | *H*(2) = 2.21 | .33 |  |
|  | eHLQ 2: Understanding of health concepts and language | 99 | *H*(2) = 0.72 | .70 |  |
|  | eHLQ 3: Ability to actively engage with digital services | 99 | *H*(2) = 1.29 | .53 |  |
|  | eHLQ 4: Feel safe and in control | 99 | *H*(2) = 0.66 | .72 |  |
|  | eHLQ 5: Motivated to engage with digital services | 99 | *H*(2) = 0.55 | .76 |  |
|  | Intention to quit smoking:  < 1 month, > 1 month | 99 | *χ^2^*(2) = 1.55 | .46 | Cramér's *V* = .13 |
|  | FTND: Nicotine dependence | 99 | *F*(2, 96) = 0.12 | .89 | *η^2^* = .002 |
|  | Intention to become sufficiently physically active:  < 1 month, > 1 month | 99 | *χ^2^*(2) = 3.14 | .21 | Cramér's *V* = .18 |
|  | GSLTPAQ: Physical activity level | 99 | *H*(2) = 1.19 | .55 |  |
| **Satisfaction with PF** | Age | 77 | Pearson *r* = 0.01 | .93 |  |
|  | Socioeconomic position:  low, middle, high | 76 | *F*(2, 73) = 2.94 | .059 | *η^2^* = .08 |
|  | eHLQ 1: Using technology to process health information | 77 | Spearman’s *ρ* = -0.16 | .18 |  |
|  | eHLQ 2: Understanding of health concepts and language | 77 | Spearman’s *ρ* = -0.02 | .87 |  |
|  | eHLQ 3: Ability to actively engage with digital services | 77 | Spearman’s *ρ* = -0.15 | .19 |  |
|  | eHLQ 4: Feel safe and in control | 77 | Spearman’s *ρ* = 0.08 | .48 |  |
|  | eHLQ 5: Motivated to engage with digital services | 77 | Spearman’s *ρ* = -0.06 | .63 |  |
|  | FTND: Nicotine dependence | 77 | Pearson *r* = -0.04 | .73 |  |
|  | GSLTPAQ: Physical activity level | 77 | Spearman’s *ρ* = -0.10 | .40 |  |
| **Usability of PF (SUS)** | Age | 77 | Spearman’s *ρ* = -0.03 | .77 |  |
|  | Socioeconomic position:  low, middle, high | 76 | *H*(2) = 1.44 | .49 |  |
|  | eHLQ 1: Using technology to process health information | 77 | Spearman’s *ρ* = 0.01 | .92 |  |
|  | eHLQ 2: Understanding of health concepts and language | 77 | Spearman’s *ρ* = 0.11 | .35 |  |
|  | eHLQ 3: Ability to actively engage with digital services | 77 | Spearman’s *ρ* = -0.05 | .70 |  |
|  | eHLQ 4: Feel safe and in control | 77 | Spearman’s *ρ* = 0.22 | .050 |  |
|  | eHLQ 5: Motivated to engage with digital services | 77 | Spearman’s *ρ* = 0.08 | .50 |  |
|  | FTND: Nicotine dependence | 77 | Spearman’s *ρ* = 0.07 | .55 |  |
|  | GSLTPAQ: Physical activity level | 77 | Spearman’s *ρ* = -0.09 | .47 |  |
| **Acceptance of the virtual coach** | Age | 77 | Pearson *r* = 0.09 | .45 |  |
|  | Socioeconomic position:  low, middle, high | 76 | *F*(2, 73) = 2.33 | .11 | *η^2^* = .06 |
|  | eHLQ 1: Using technology to process health information | 77 | Spearman’s *ρ* = 0.002 | .99 |  |
|  | eHLQ 2: Understanding of health concepts and language | 77 | Spearman’s *ρ* = 0.07 | .58 |  |
|  | eHLQ 3: Ability to actively engage with digital services | 77 | Spearman’s *ρ* = -0.12 | .31 |  |
|  | eHLQ 4: Feel safe and in control | 77 | Spearman’s *ρ* = 0.15 | .20 |  |
|  | eHLQ 5: Motivated to engage with digital services | 77 | Spearman’s *ρ* = 0.08 | .47 |  |
|  | FTND: Nicotine dependence | 77 | Pearson *r* = 0.14 | .22 |  |
|  | GSLTPAQ: Physical activity level | 77 | Spearman’s *ρ* = -0.11 | .33 |  |
| *Note.*  PF = Perfect Fit; eHLQ = eHealth Literacy Questionnaire; FTND = Fagerström Test for Nicotine Dependence; GSLTPAQ = Godin-Shephard Leisure-Time Physical Activity questionnaire; SUS = System Usability Scale.  **P* < .05; ***P* < .01. | | | | | |

**Additional results: Feasibility of conducting the study**

| **Table S4**  *Number and percentage of participants enrolled through each recruitment strategy.* | |
| --- | --- |
| **Recruitment strategy** | ***n* (%)** |
| Newsletter or magazine of health insurance provider | 37 (37%) |
| Social media (Facebook, Instagram, or LinkedIn) | 21 (21%) |
| Family, friends, or work contacts | 21 (21%) |
| Flyer handed out in person by a researcher | 4 (4%) |
| Invitation after participation in previous LUMC research | 4 (4%) |
| Article in local newspaper (Leidsch Dagblad) | 4 (4%) |
| General practitioner’s practice | 2 (2%) |
| Announcement on the Perfect Fit study website | 2 (2%) |
| Paper flyer seen at a public location | 1 (1%) |
| Other | 3 (3%) |
| Missing | 1 (1%) |

**References**

1. Meijer E, Gebhardt WA, Van Laar C, Kawous R, Beijk SCAM. Socio-economic status in relation to smoking: The role of (expected and desired) social support and quitter identity. Soc Sci Med. 2016 Aug;162:41-9. PMID: 27328056. doi: 10.1016/j.socscimed.2016.06.022.

2. Tukey JW. The future of data analysis. Breakthroughs in Statistics: Methodology and Distribution: Springer; 1962. p. 408-52.

3. To QG, Green C, Vandelanotte C. Feasibility, usability, and effectiveness of a machine learning-based physical activity chatbot: quasi-experimental study. Jmir Mhealth Uhealth. 2021 Nov 2;9(11). PMID: 34842552. doi: 10.2196/28577.

4. Braem CIR, Yavuz US, Hermens HJ, Veltink PH. Missing data statistics provide causal insights into data loss in diabetes health monitoring by wearable sensors. Sensors-Basel. 2024 Mar;24(5). PMID: 38475061. doi: 10.3390/s24051526.
