# Supplementary material for: Evaluation of the Feasibility and Acceptability of Perfect Fit, a Virtual Coach–Based mHealth Intervention for Smoking Cessation and Physical Activity in Adults: Mixed Methods Study
Source: JMIR Hum Factors. 2026 Jul 14;13:e83456. doi: 10.2196/83456 (PMC13367948; doi:10.2196/83456)
Supplement: Multimedia Appendix 3 [file humanfactors-v13-e83456-s003.docx]

**Appendix 3.** *Sensor connection issues: Technical lessons learned and recommendations*

**Summary of sensor connection issues, troubleshooting efforts, and recommendations**

***Observed issues***

- Background app closure: The sensor data collector app was sometimes shut down by the smartphone operating system, interrupting data transfer. The app needed to be active at least once every 24 hours to maintain the connection.
- Discontinued participation: Some participants possibly stopped wearing the smartwatch without notifying the research team, which may explain part of the missing data.
- Unidentified technical errors: In some cases, devices remained connected but failed to transfer data reliably. We suspect that the smartwatch occasionally froze due to communication requests from the phone, resulting in inaccurate or missing data.

***Troubleshooting efforts***

- Instructing users to regularly open the sensor data collector app, which may have reduced issues, though this could not be definitively confirmed.
- Checking relevant smartphone and smartwatch settings that could potentially affect data transfer (e.g., Bluetooth status, Wi-Fi connection, battery-saving mode) in phone calls with participants.
- Assisting participants in resetting the smartwatch widget remotely. This was temporarily effective but time-consuming and not a durable solution.

***Recommendations for future researchers***

- Anticipate technical issues and plan solutions proactively.
- Conduct extensive pilot testing on different device types both prior to and during the study to identify issues early. Piloting with participants from the target population and across different phone operating systems is also valuable, as practical issues may not be detected by research staff alone.
  - We extensively tested the data collection setup for two weeks using two different android devices and two iOS devices and captured no significant issues. This has been insufficient to detect sensor data issues experienced during the intervention.
- Implement real-time monitoring systems to detect missing data and allow for rapid intervention, including fallback methods to maintain system functionality when data are temporarily unavailable (e.g., automatic alerts to researchers or support staff when data is missing for a certain period; or using the last recorded value to continue the intervention until the next data point is received).
- Minimize technical complexity, considering that many users have limited technical literacy.
- In this study, multiple apps were used to deliver the intervention, which resulted in connectivity issues and increased complexity. Aim to consolidate functionalities into a single app to reduce connectivity barriers, improve user-friendliness, and minimize battery consumption.
- Establish a clear workflow for interdisciplinary communication and appoint accessible support staff who can assist participants with technical issues. Building rapport with participants and providing approachable, ongoing support can increase participants’ motivation to persevere when facing technical difficulties.
