# Supplementary material for: Evaluation of the Feasibility and Acceptability of Perfect Fit, a Virtual Coach–Based mHealth Intervention for Smoking Cessation and Physical Activity in Adults: Mixed Methods Study
Source: JMIR Hum Factors. 2026 Jul 14;13:e83456. doi: 10.2196/83456 (PMC13367948; doi:10.2196/83456)
Supplement: Checklist 1 [file humanfactors-v13-e83456-s004.docx]

*Good Reporting of A Mixed Methods Study (GRAMMS) checklist*

| **GRAMMS item** | **Location in manuscript** |
| --- | --- |
| Describe the justification for using a mixed methods approach to the research question | Introduction, final paragraph, p. 4 |
| Describe the design in terms of the purpose, priority, and sequence of methods | Methods, Study design section, p. 5 |
| Describe each method in terms of sampling, data collection, and analysis | Methods, Participants and recruitment, Procedures, and Data analysis sections, pp. 5–7 and 9–10 |
| Describe where integration has occurred, how it has occurred, and who has participated in it | Methods, Data analysis – Qualitative and Data triangulation sections, p. 10 |
| Describe any limitations of one method associated with the presence of the other method | Discussion, Strengths and limitations section, pp. 23 |
| Describe any insights gained from mixing or integrating methods | Discussion, pp. 21–24 |

**Reference**

O'Cathain A, Murphy E, Nicholl J. The quality of mixed methods studies in health services research. *J Health Serv Res Po*. 2008 Apr;13(2):92-8. PMID: 18416914. doi: 10.1258/jhsrp.2007.007074.
